# Hydrogen‐Mediated Activation of the Nrf2/HO‐1 Signaling Pathway Improves Cognitive Impairment in Sleep‐Deprived Mice

**DOI:** 10.1002/cns.70770

**Published:** 2026-02-01

**Authors:** QiFan Xiao, ShiRui Zhou, Bin Tang, YuQing Zhu

**Affiliations:** ^1^ General Practice International Department of China‐Japan Friendship Hospital Beijing China; ^2^ Graduate School Beijing University of Chinese Medicine Beijing China

**Keywords:** cognitive impairment, hydrogen, neuroinflammation, Nrf2/HO‐1 signaling pathway, oxidative stress, sleep deprivation

## Abstract

**Aims:**

This study investigated the effects of hydrogen (H_2_) on cognitive impairment in sleep‐deprived mice mediated by the nuclear factor erythroid 2‐related factor 2 (Nrf2)/heme oxygenase‐1 (HO‐1) signaling pathway.

**Methods:**

A chronic sleep deprivation (SD) model was established using the modified multiple platform method, with 18 h of deprivation daily for 28 consecutive days. Cognitive function was evaluated using the Morris water maze and novel object recognition (NOR) test. Histopathological and biochemical analyses, including hematoxylin and eosin staining, Nissl staining, immunohistochemistry, and enzyme‐linked immunosorbent assay, were performed to assess oxidative stress and inflammation‐related markers.

**Results:**

The results demonstrated that H_2_‐treated mice showed significantly shorter escape latency, longer target quadrant duration, and higher NOR index compared to controls. Concurrently, Nrf2/HO‐1 expression was significantly upregulated, while interleukin‐1β and tumor necrosis factor‐alpha levels were reduced. Additionally, glutathione peroxidase and superoxide dismutase activities were restored.

**Conclusion:**

These results indicate that H_2_ alleviates oxidative stress and neuroinflammation through Nrf2/HO‐1 pathway activation, mitigating SD‐induced cognitive impairment. This research provides a theoretical foundation for potential clinical applications.

## Graphical abstract

Hydrogen enhances memory and learning capabilities in sleep‐deprived mice by activating the Nrf2/HO‐1 signaling pathway, while suppressing neuronal damage, oxidative stress, and inflammatory responses.

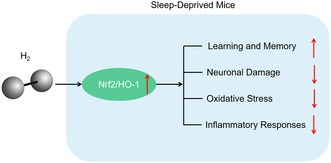

## 1 Introduction

The increasingly rapid pace of modern life has resulted in widespread sleep deprivation (SD), clinically defined as obtaining fewer than 4 h of sleep per day, representing a considerable public health concern. Growing data show that SD adversely influences numerous cognitive areas, especially impairing learning capacity, attention, and memory performance [1, 2, 3, 4]. Furthermore, epidemiological investigations have revealed robust associations between prolonged SD and the onset of diverse conditions, such as cardiometabolic disorders, cancers, autoimmune diseases, and neurodegenerative pathologies [5]. The substantial clinical incidence of SD poses a serious risk to population well‐being [6]. Nevertheless, effective interventions remain scarce. Hence, elucidating the mechanisms responsible for SD‐induced learning and memory deficits, and identifying new preventive and treatment strategies, is critically important.

As a brain region essential for higher cognitive operations—including emotional control, learning, and memory—the hippocampus is particularly susceptible to SD. Prolonged sleep disturbance may induce progressive harmful consequences, reducing hippocampal cell proliferation, survival, and neurogenesis, which eventually undermines neurobehavioral capacities such as mood, cognition, and memory [7, 8, 9]. SD can also disrupt hippocampal function by impairing synaptic plasticity at electrophysiological, molecular, and structural levels [10, 11]. The cognitive and memory impairments arising from SD are predominantly mediated by oxidative injury and neuroinflammatory processes [12, 13]. Therefore, addressing oxidative stress and inflammation constitutes a viable approach for mitigating SD‐related cognitive decline.

The nuclear factor erythroid 2‐related factor 2 (Nrf2)/heme oxygenase‐1 (HO‐1) signaling cascade plays a pivotal role in modulating oxidative stress and inflammatory reactions [14]. Hydrogen (H_2_), functioning as an antioxidant, can stimulate the Nrf2/HO‐1 pathway, offering defense against multiple forms of neurological damage [15]. Over the past 10 years, molecular H_2_ has emerged as a promising therapeutic candidate with broad potential advantages, including control of proinflammatory mediators [16] and improvement of insulin sensitivity [17]. Notably, H_2_ has no documented cytotoxic impact even at high concentrations, making it a safe treatment with few side effects [18]. It has been shown to diminish oxidative damage, reinforce immune pathways, alleviate chemical and physiological stressors, and ameliorate metabolic disturbances [19]. Previous research indicates that H_2_ inhalation enhances sleep quality and cognitive performance in subjects with sleep problems [20], supporting the notion that H_2_ may deliver these benefits via its antioxidant characteristics [21]. The present investigation seeks to evaluate this hypothesis using animal experiments, thereby laying a theoretical groundwork for clinical H_2_ inhalation therapy.

## 2 Materials and Methods

### 2.1 Animal

All procedures involving animals were approved by the Institutional Animal Care and Use Committee of the China‐Japan Friendship Hospital. Male and female C57BL/6 mice (6 weeks old) were obtained from Changzhou Cavens Laboratory Animal Co. Ltd (China). Mice were maintained in a regulated setting (24°C ± 1°C, 12 h light/12 h dark cycle with illumination from 7:00 a.m. to 7:00 p.m.) and allowed free access to food and water. Animals were habituated for 1 week prior to experiments.

### 2.2 SD Mouse Model

The SD model was generated via a modified multiple‐platform technique [22]. Briefly, six mice per group were placed in polypropylene cages (41 × 34 × 16 cm) containing 12 platforms (3 cm diameter, 5 cm height) submerged in water to a depth 1 cm below the platform surfaces. Animals could move freely by jumping among platforms. Food and water were continuously accessible.

### 2.3 Experimental Groups

Sixty mice were randomly divided into six groups (*n* = 10 per group, sexes balanced). Group details and treatment regimens are shown in Table 1. Short hairpin RNA (shRNA) lentiviral vectors directed against Nrf2 (sh‐Nrf2) and corresponding negative control viruses (sh‐NC) were sourced from GeneChem (China).

**TABLE 1:** Experimental grouping and treatments in mice.

Group	Treatment protocol
Control	Mice without sleep deprivation
Model	Mice subjected to sleep deprivation using the modified multiple platform method (18 h/day for 28 consecutive days)
H_2_	After 21 days of sleep deprivation, mice received 33.3% O_2_ + 66.7% H_2_ inhalation (2 sessions/day, 3 h/session) for 7 days with concurrent sleep deprivation
O_2_	After 21 days of sleep deprivation, mice received 33.3% O_2_ + 66.7% N_2_ inhalation (2 sessions/day, 3 h/session) for 7 days with concurrent sleep deprivation
H_2_ + sh‐NC	After 21 days of sleep deprivation, mice received stereotaxic injection of sh‐NC lentivirus followed by 7 days of 33.3% O_2_ + 66.7% H_2_ inhalation (2 sessions/day, 3 h/session) with concurrent sleep deprivation
H2 + sh‐Nrf2	After 21 days of sleep deprivation, mice received stereotaxic injection of sh‐Nrf2 lentivirus followed by 7 days of 33.3% O_2_ + 66.7% H_2_ inhalation (2 sessions/day, 3 h/session) with concurrent sleep deprivation

### 2.4 Morris Water Maze Test

A black cylindrical pool (150 cm diameter, 50 cm height) was used. The tank was notionally separated into four quadrants (I–IV). A concealed circular platform (10 cm diameter) was placed 1 cm under the water surface in quadrant I's center. Fixed visual cues were positioned around the pool. Water was rendered opaque with white dye. Testing comprised two phases: navigation and spatial probe.

Navigation phase: Mice received 4 trials daily for 5 successive days (1‐h intervals between trials). Escape latency (time from entering water at varying start locations to finding the platform) was recorded. The mean daily value from four trials was analyzed.

Spatial probe phase: On Day 6, the platform was withdrawn. Mice were released from the quadrant opposite the original platform site. Their swimming path over 60 s was tracked, and time spent in the target quadrant and platform crossings were quantified.

### 2.5 Novel Object Recognition (NOR) Test

Two identical objects were securely placed in an open arena with double‐sided tape, positioned 6 cm from adjacent walls in two non‐release corners to prevent displacement. All sessions were video‐recorded and analyzed with behavior‐tracking software.

Day 1 (Training): Mice were brought to the testing room and acclimatized for ≥ 30 min. Each mouse was placed facing a release‐corner wall and permitted to freely explore the arena and objects for 5 min. After a 20‐min inter‐trial interval, the arena was cleaned thoroughly with 70% ethanol to remove odor cues.

Day 2 (Testing): One familiar object was exchanged for a novel object, fixed similarly. Each mouse was again introduced facing the wall and given 5 min exploration. The NOR index was computed as follows:

$$\begin{array}{c} \left[\left(\text{Time exploring novel object}-\text{Time exploring familiar object}\right)/\left(\text{Total exploration time}\right)\right]\times 100\%. \end{array}$$

### 2.6 Hematoxylin and Eosin (H&E) Staining

Following behavioral assessments, brain samples were collected after euthanasia. Tissues were fixed in 4% paraformaldehyde, embedded in paraffin, and sliced into 5 μm sections. Hippocampal sections were incubated overnight at 60°C, then deparaffinized through three 20‐min xylene washes. Rehydration was accomplished via a descending ethanol series (100%, 95%, 85%, 75%, 5 min each) followed by rinsing with distilled water. Staining was carried out using a commercial H&E kit (Abiowell, China), and sections were inspected under a light microscope (BA210T, Motic, China).

### 2.7 Nissl Staining

After routine deparaffinization and rehydration, tissue sections were treated with Nissl staining solution for 30–60 s. Excess stain was removed by washing with distilled water, followed by differentiation in 1% glacial acetic acid. Processed sections were coverslipped with buffered glycerol mounting medium and examined by light microscopy.

### 2.8 Enzyme‐Linked Immunosorbent Assay (ELISA)

Brain tissue samples were mechanically homogenized and stored at −20°C for 24 h. After two complete freeze–thaw cycles at 2°C–8°C to disrupt cells, homogenates were centrifuged at 5000×*g* for 5 min to collect supernatant. ELISA was conducted following kit instructions. Commercial kits were utilized to detect cytokines: mouse tumor necrosis factor‐α (TNF‐α; KE10002), interleukin (IL)‐1β (KE10003), and IL‐6 (KE10007) from Proteintech (USA), as well as oxidative stress markers glutathione peroxidase (GSH‐PX, A005‐1), malondialdehyde (MDA, A003‐1), and superoxide dismutase (SOD, A003‐1) from Nanjing Jiancheng Bioengineering Institute (China).

### 2.9 Immunohistochemistry

Following standard deparaffinization and rehydration steps, tissue sections underwent antigen retrieval by heating in 0.01 M citrate buffer (pH 6.0) followed by gradual cooling to ambient temperature. After three 3‐min washes with phosphate‐buffered saline (PBS, 0.01 M), endogenous peroxidase activity was blocked with 1% periodic acid solution (10 min incubation) and subsequent PBS rinses (3 × 3 min). Primary antibody incubation occurred at 4°C for 12–16 h using these antibodies (all from Proteintech): postsynaptic density protein 95 (PSD95, 1:200), synaptophysin (SYN, 1:200), Nrf2 (1:200), and HO‐1 (1:200). Secondary detection involved 30‐min incubation at 37°C with horseradish peroxidase‐conjugated anti‐rabbit immunoglobulin G (IgG). Color development employed a 3,3′‐diaminobenzidine substrate system (ZSGB‐BIO, China). Final processing included sequential dehydration in graded ethanols (60–100%, 5 min per concentration), xylene clearing (10 min), and mounting with neutral gum for light microscopic assessment.

### 2.10 Preparation of Hydrogen‐Rich Saline

Hydrogen‐rich saline was generated according to a published method [23]. Briefly, hydrogen gas was dissolved into saline under high pressure (0.4 MPa) for 2 h using specialized equipment to attain supersaturation. Hydrogen concentration was determined via gas chromatography (Biogas Analyzer Systems‐1000, Mitleben, Japan).

### 2.11 Cell Culture and Treatment

PC12 cells were cultivated under standard culture conditions [24]. Cells were grown in RPMI‐1640 medium at 37°C in a humidified incubator with 5% CO_2_, with medium refreshed every 2 days. An in vitro neuronal injury model was produced by exposing PC12 cells to 200 μM corticosterone (CORT) for 24 h [25]. To investigate the influence of H_2_ on neuronal injury, cells were treated with various concentrations (37.5–300 μM) of hydrogen‐rich saline for 24 h.

### 2.12 Cell Viability Assay

Viability was determined using a CCK‐8 kit (Beyotime, China) as per manufacturer's instructions. PC12 cells were seeded in a 96‐well plate and cultured for 72 h. Then, 10 μL of CCK‐8 solution was introduced into each well. After 2 h incubation, absorbance at 450 nm was recorded.

### 2.13 Cell Apoptosis Assay

Apoptosis was evaluated with an Annexin V‐FITC/PI Apoptosis Detection Kit (Keygen, China) following the manufacturer's protocol. Cells were resuspended in binding buffer and stained with Annexin V‐FITC and propidium iodide (PI) in darkness for 20 min. Apoptotic rate was then assessed by flow cytometry (BD FACS Aria; BD Biosciences, USA).

### 2.14 Quantitative Real‐Time Polymerase Chain Reaction (RT‐qPCR)

Total RNA was isolated with TRIzol (Invitrogen, USA). cDNA was synthesized using the MonScript RT III Kit (Monad). Gene expression analysis was performed on a real‐time PCR system (Thermo Fisher Scientific, USA) employing SYBR Green chemistry (Monad). Relative quantification was determined via the $2^{-\Delta \Delta C_{t}}$ method, using glyceraldehyde‐3‐phosphate dehydrogenase (GAPDH) as the reference gene. Primer sequences are provided in Table 2.

**TABLE 2:** Primer sequences for RT‐qPCR.

Gene	Primer sequence (5′ → 3′)
Nrf2	F: AAAATCATTAACCTCCCTGTTGAT
	R: CGGCGACTTTATTCTTACCTCTC
HO‐1	F: CAAGCCGAGAATGCTGAGTTCATG
	R: GCAAGGGATGATTTCCTGCCAG
GAPDH	F: CATCAACGGGAAGCCCATC
	R: CTCGTGGTTCACACCCATC

### 2.15 Western Blot

Total protein was extracted with radioimmunoprecipitation assay lysis buffer (Solarbio, China) and quantified with a bicinchoninic acid assay kit (Solarbio). Equal protein amounts were separated by 12% sodium dodecyl sulfate‐polyacrylamide gel electrophoresis and transferred to polyvinylidene fluoride membranes (Solarbio). After blocking, membranes were incubated overnight at 4°C with primary antibodies: anti‐Nrf2 (1:1000, Invitrogen), anti‐HO‐1 (1:2000, Abcam), and anti‐GAPDH (1:5000, Proteintech). Subsequently, membranes were incubated for 1 h with a horseradish peroxidase‐conjugated goat anti‐rabbit IgG secondary antibody (1:5000, Proteintech). Protein bands were visualized using enhanced chemiluminescence reagent (Invitrogen) and captured with a chemiluminescence detection system (Bio‐Rad, USA). Band intensity was quantified with ImageJ software (National Institutes of Health, USA).

### 2.16 Statistical Analysis

Statistical analyses were conducted using GraphPad Prism 10 software (GraphPad Software Inc., USA). Data are expressed as mean ± standard deviation. Comparisons between two groups were analyzed by Student's *t*‐test, while multiple‐group comparisons were evaluated by one‐way analysis of variance followed by Tukey's post hoc test. A *p*‐value < 0.05 was deemed statistically significant.

## 3 Results

### 3.1 H_2_ Treatment Improves Learning and Memory in Sleep‐Deprived Mice

**FIGURE 1:**
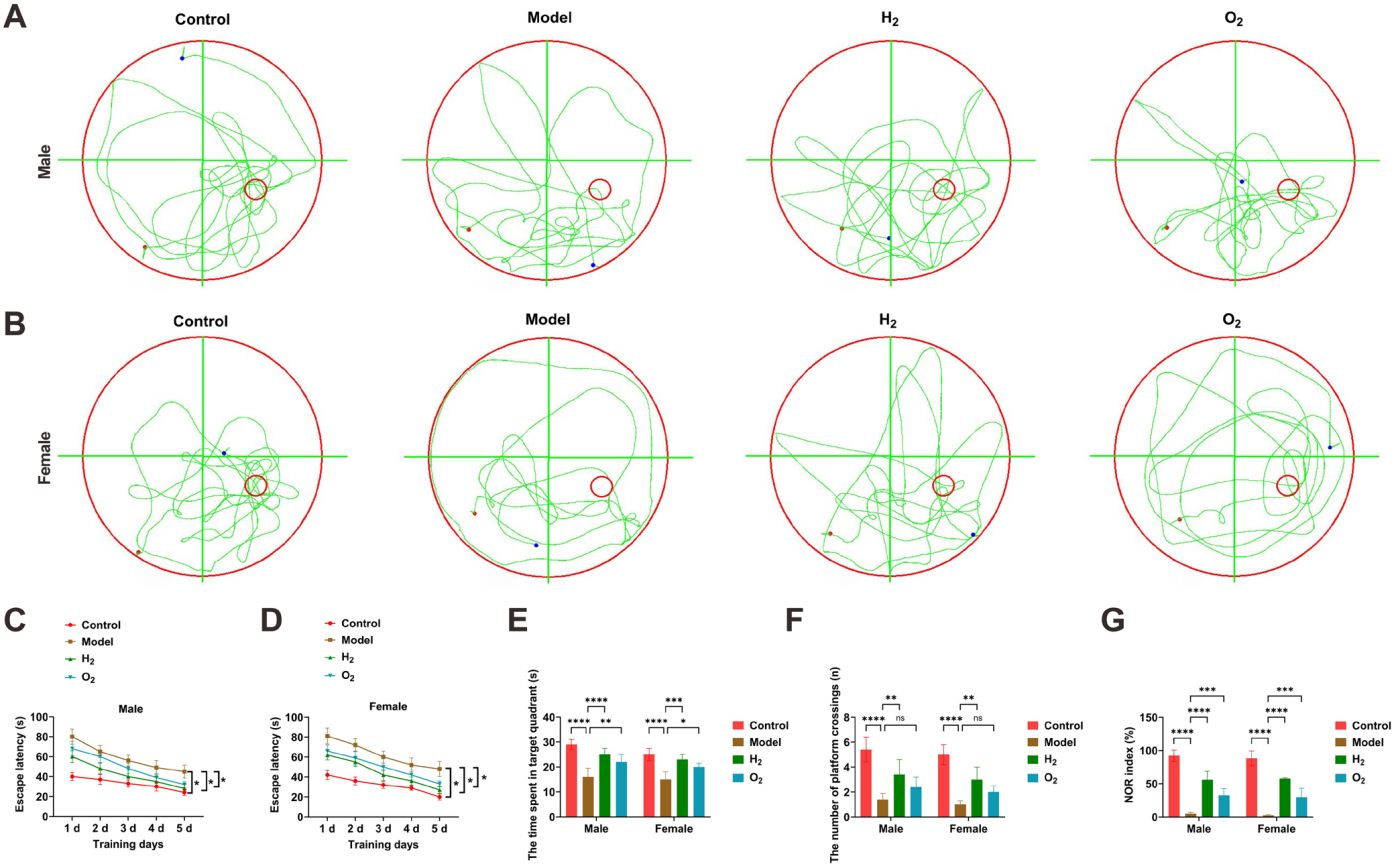
H_2_ treatment improves learning and memory in sleep‐deprived mice. (A, B) Morris water maze test results; (C, D) escape latency time; (E) time spent in target quadrant; (F) number of platform crossings; (G) NOR index. Data presented as mean ± SD. *p < 0.05, **p < 0.01, ***p < 0.001, ****p < 0.0001.

The Morris water maze was employed to evaluate learning and memory among experimental groups (Figure 1A,B). Analysis of escape latencies indicated that SD mice in the model group had considerably longer latencies compared with control animals during training (Figure 1C,D). H_2_ treatment substantially shortened these latencies (Figure 1C,D). In the spatial probe test, model group mice spent less time in the target quadrant and performed fewer platform crossings within 60 s after platform removal relative to controls (Figure 1E,F). These impairments were significantly reversed by H_2_ administration (Figure 1E,F). The NOR test showed a markedly lower NOR index in the model group versus controls, which was notably restored after H_2_ intervention (Figure 1G).

### 3.2 H_2_ Therapy Ameliorates Hippocampal Neuronal Pathological Damage in Sleep‐Deprived Mice

**FIGURE 2:**
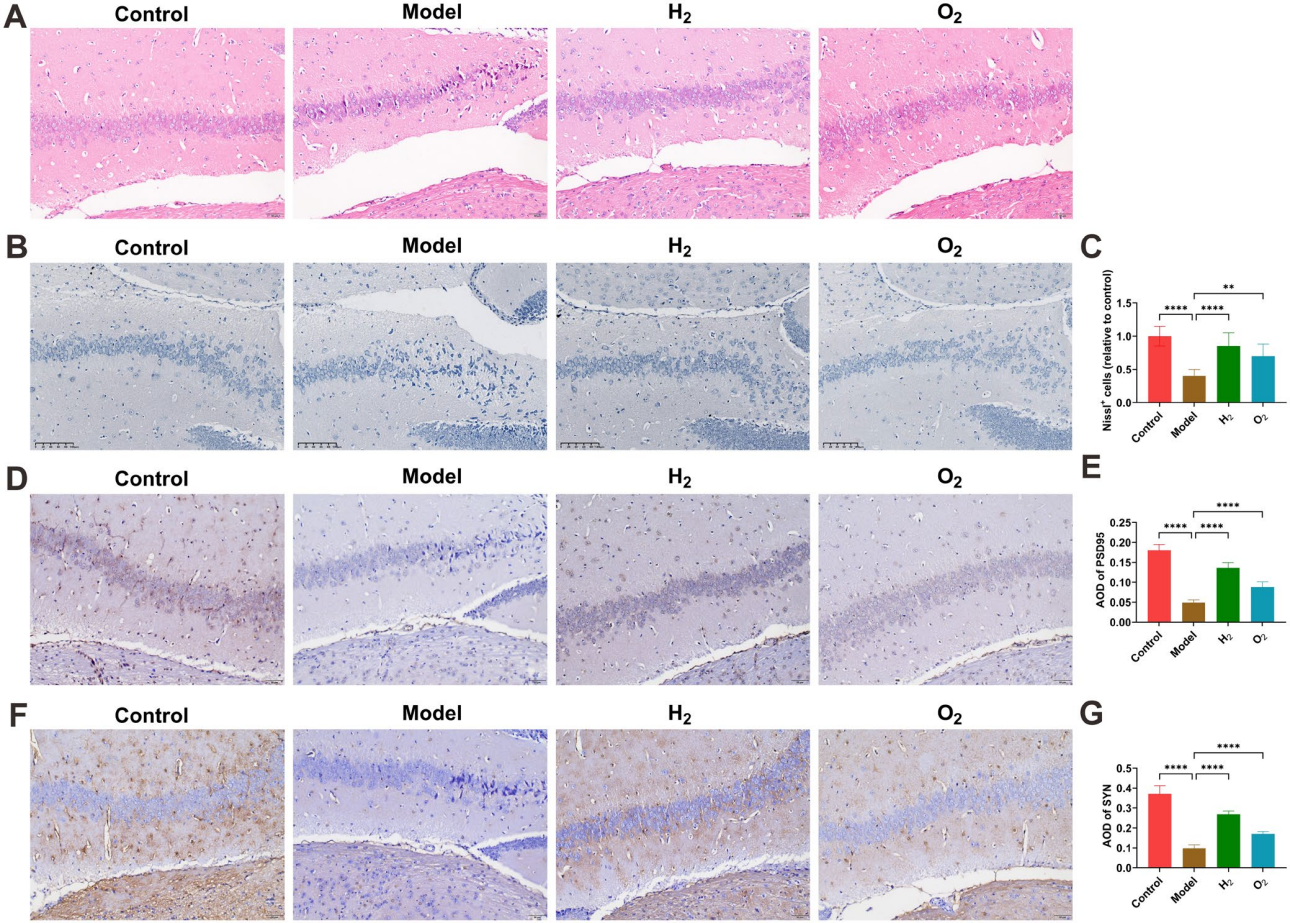
H_2_ therapy ameliorates hippocampal neuronal pathological damage in sleep‐deprived mice. (A) H&E staining of hippocampal CA1 morphology; (B, C) Nissl staining showing intact neuron counts in CA1 region; (D, E) immunohistochemical analysis of PSD95 expression; (F, G) immunohistochemical analysis of SYN expression. Data presented as mean ± SD. *p < 0.05, **p < 0.01, ***p < 0.001, ****p < 0.0001.

Relative to controls, the model group exhibited disorganized hippocampal neuron alignment and condensed nuclei. H_2_ delivery significantly improved these pathological alterations (Figure 2A). Nissl staining revealed that control group neurons were orderly arranged with normal morphology, evenly distributed Nissl bodies, and higher neuronal density. By contrast, the model group displayed disordered neuronal arrangement, substantially reduced cell numbers, and evident necrotic and pyknotic changes. H_2_ intervention significantly raised neuronal counts and Nissl body density while restoring normal neuronal morphology and diminishing cellular injury (Figure 2B,C). To examine whether hippocampal pathology affects synaptic transmission, synaptic protein expression (PSD95 and SYN) in the CA1 region was assessed via immunohistochemistry. The model group showed significantly lower PSD95 and SYN expression compared with controls, whereas H_2_ treatment effectively recovered their expression levels (Figure 2D–G).

### 3.3 H_2_ Therapy Attenuates Oxidative Stress and Inflammatory Responses in Sleep‐Deprived Mice

**FIGURE 3:**
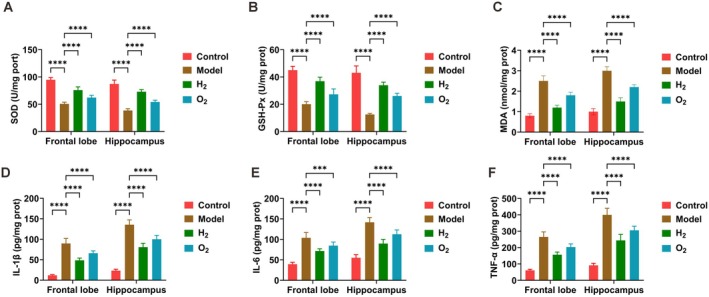
H_2_ therapy attenuates oxidative stress and inflammatory responses in sleep‐deprived mice. (A–C) SOD, GSH‐Px and MDA levels measured by commercial kits; (D–F) IL‐1β, IL‐6, and TNF‐α concentrations determined by ELISA. Data presented as mean ± SD. *p < 0.05, **p < 0.01, ***p < 0.001, ****p < 0.0001.

In brain tissues, activities of antioxidant enzymes (SOD, GSH‐Px) and levels of the lipid peroxidation marker MDA were measured. Compared with the control group, sleep‐deprived mice exhibited significantly reduced SOD and GSH‐Px activities together with elevated MDA content. H_2_ treatment effectively reversed these changes, restoring antioxidant enzyme activities and lowering lipid peroxidation (Figure 3A–C). Subsequently, concentrations of pro‐inflammatory cytokines (IL‐1β, IL‐6, and TNF‐α) were evaluated. Sleep‐deprived animals displayed substantially raised levels of all inflammatory mediators relative to controls, while H_2_ administration significantly suppressed these pro‐inflammatory responses (Figure 3D–F).

### 3.4 H_2_ Therapy Activates the Nrf2/HO‐1 Signaling Pathway

**FIGURE 4:**
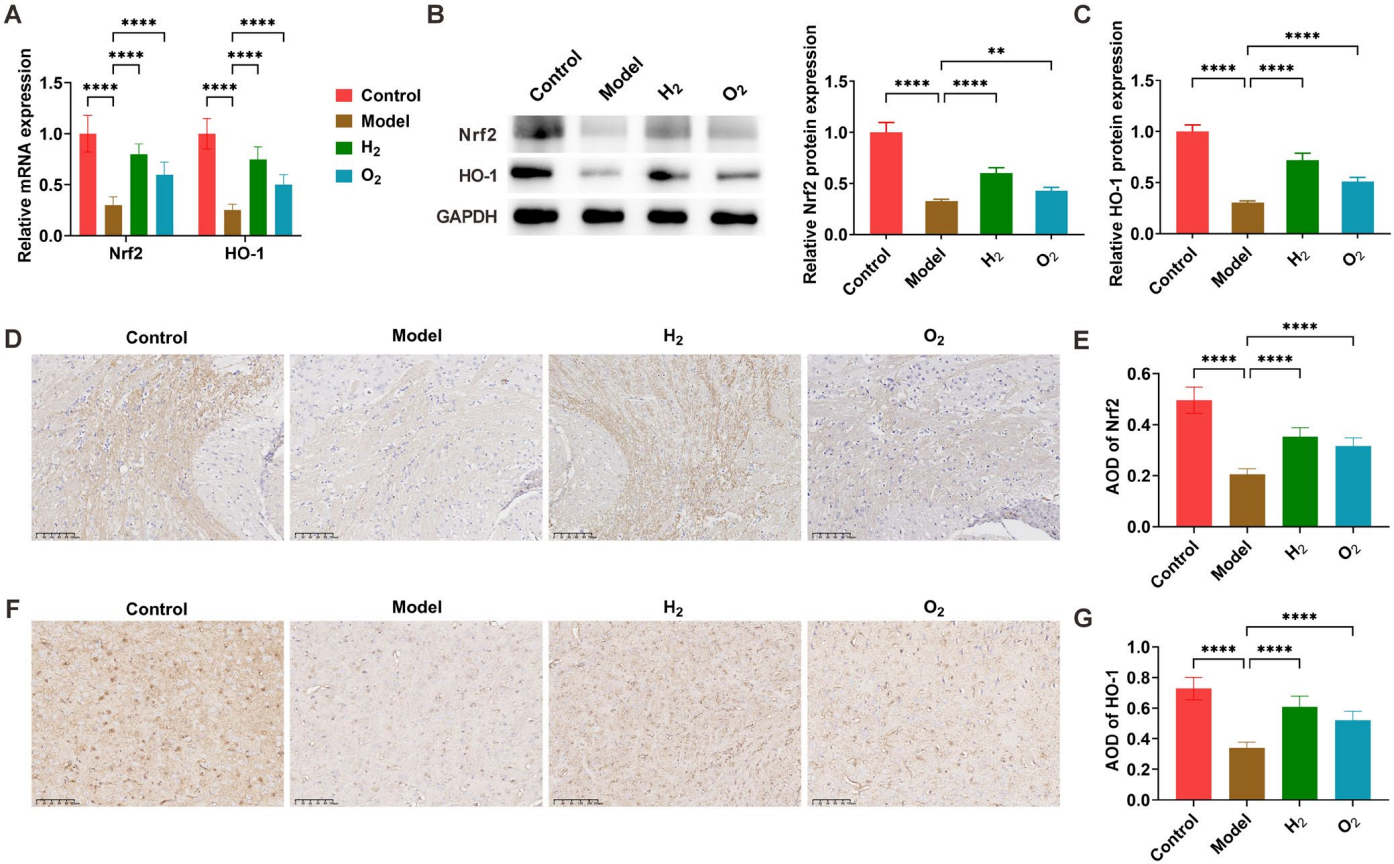
H_2_ therapy activates the Nrf2/HO‐1 signaling pathway. (A–C) Nrf2 and HO‐1 expression analyzed by RT‐qPCR and Western blot; (D, E) immunohistochemical detection of Nrf2; (F, G) immunohistochemical detection of HO‐1. Data presented as mean ± SD. *p < 0.05, **p < 0.01, ***p < 0.001, ****p < 0.0001.

Nrf2 and HO‐1 expression in brain tissues was analyzed by RT‐qPCR and Western blot. Relative to controls, the model group showed significantly down‐regulated Nrf2 and HO‐1 expression at both mRNA and protein levels. H_2_ treatment markedly upregulated Nrf2 and HO‐1 expression (Figure 4A–C). Immunohistochemical analysis confirmed these results, demonstrating significantly decreased Nrf2 and HO‐1 protein expression in the model group compared with controls. H_2_ intervention effectively reinstated Nrf2 and HO‐1 protein levels (Figure 4D–G).

### 3.5 Nrf2 Knockdown Attenuates H_2_‐Induced Activation of the Nrf2/HO‐1 Pathway

**FIGURE 5:**
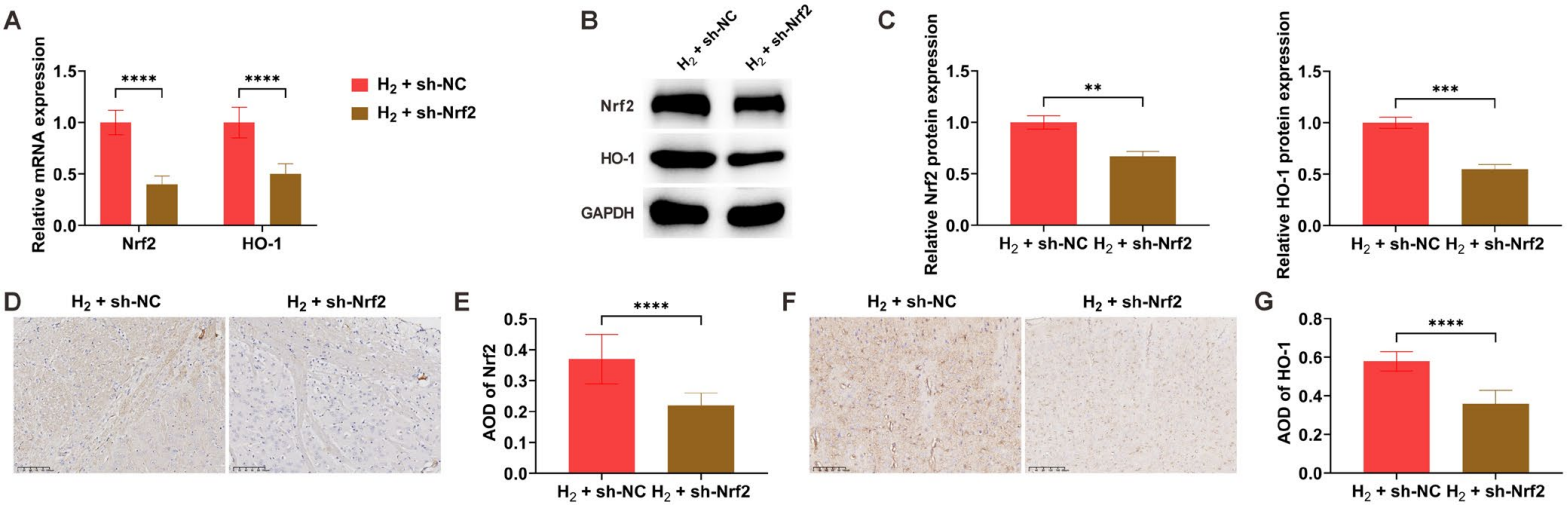
Nrf2 knockdown attenuates H_2_‐induced activation of the Nrf2/HO‐1 pathway. (A–C) Nrf2 and HO‐1 expression analyzed by RT‐qPCR and Western blot; (D) immunohistochemical detection of Nrf2; (D, E) immunohistochemical detection of Nrf2; (F, G) immunohistochemical detection of HO‐1. Data presented as mean ± SD. *p < 0.05, **p < 0.01, ***p < 0.001, ****p < 0.0001.

To further clarify the functional contribution of the Nrf2/HO‐1 pathway, Nrf2 expression was genetically suppressed. Outcomes from RT‐qPCR, Western blot, and immunohistochemical analyses consistently indicated that the H_2_‐induced upregulation of both Nrf2 and HO‐1 expression was markedly diminished following Nrf2 knockdown (Figure 5A–G).

### 3.6 Nrf2 Knockdown Attenuates H_2_‐Induced Improvement of Learning and Memory in Sleep‐Deprived Mice

**FIGURE 6:**
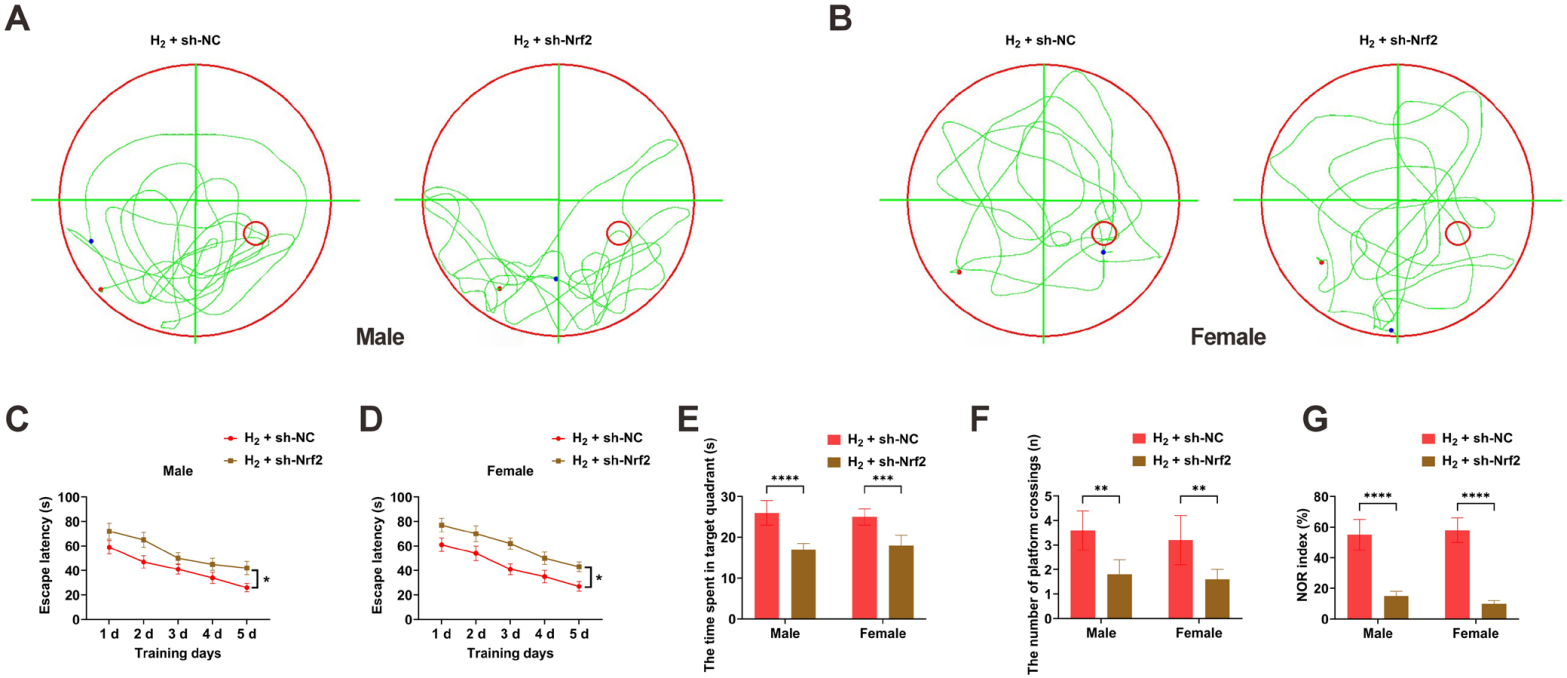
Nrf2 knockdown attenuates H_2_‐induced improvement of learning and memory in sleep‐deprived mice. (A, B) Morris water maze test results; (C, D) escape latency time; (E) time spent in target quadrant; (F) number of platform crossings; (G) NOR index. Data presented as mean ± SD. *p < 0.05, **p < 0.01, ***p < 0.001, ****p < 0.0001.

Behavioral evaluations using the Morris water maze and NOR tests demonstrated that the beneficial influences of H_2_ on learning and memory were significantly attenuated following Nrf2 knockdown in SD mice (Figure 6A–G).

### 3.7 Nrf2 Knockdown Attenuates the Neuroprotective Effects of H_2_ Therapy in Sleep‐Deprived Mice

**FIGURE 7:**
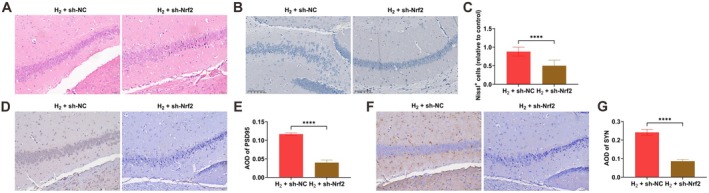
Nrf2 knockdown attenuates the neuroprotective effects of H_2_ therapy in sleep‐deprived mice. (A) H&E staining of hippocampal CA1 morphology; (B, C) Nissl staining showing intact neuron counts in CA1 region; (D, E) immunohistochemical analysis of PSD95 expression; (F, G) immunohistochemical analysis of SYN expression. Data presented as mean ± SD. *p < 0.05, **p < 0.01, ***p < 0.001, ****p < 0.0001.

Histological analysis via H&E and Nissl staining revealed that Nrf2 knockdown substantially impaired the H_2_‐mediated alleviation of hippocampal neuronal pathology in SD mice (Figure 7A–C). Moreover, immunohistochemistry indicated that the H_2_‐induced increase in synaptic proteins PSD95 and SYN in the CA1 region was notably reduced after Nrf2 knockdown (Figure 7D–G).

### 3.8 Nrf2 Knockdown Attenuates H_2_‐Mediated Amelioration of Oxidative Stress and Inflammation in Sleep‐Deprived Mice

**FIGURE 8:**
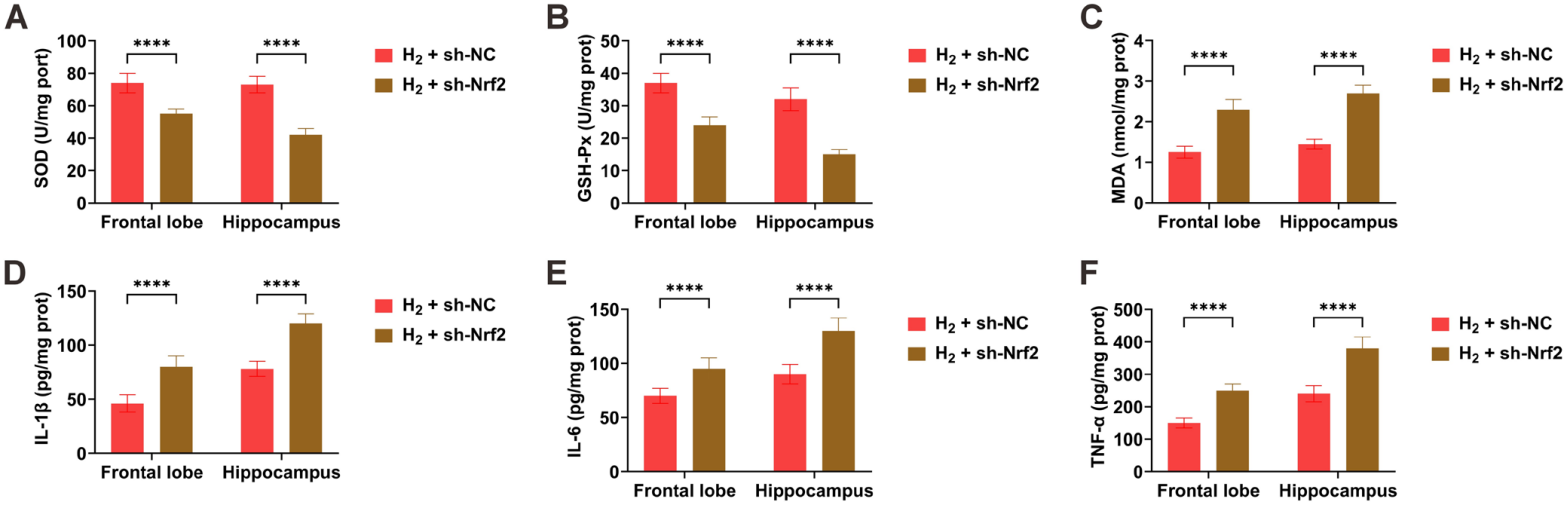
Nrf2 knockdown attenuates H_2_‐mediated amelioration of oxidative stress and inflammation in sleep‐deprived mice. (A–C) SOD, GSH‐Px, and MDA levels measured by commercial kits; (D–F) IL‐1β, IL‐6 and TNF‐α concentrations determined by ELISA. Data presented as mean ± SD. *p < 0.05, **p < 0.01, ***p < 0.001, ****p < 0.0001.

Nrf2 knockdown significantly lessened the therapeutic effects of H_2_ treatment on oxidative stress markers and inflammatory responses in brain tissues of SD mice (Figure 8A–F).

### 3.9 H_2_ Ameliorates Neuroidal Injury In Vitro

An in vitro neuronal injury model was created by treating PC12 cells with 200 μM CORT for 24 h [25]. To examine H_2_'s effect on neuronal injury in vitro, PC12 cells were exposed to varying concentrations (37.5–300 μM) of hydrogen‐rich saline for 24 h. Results showed that hydrogen‐rich saline enhanced cell viability and inhibited apoptosis in PC12 cells (Figure S1A,B). Additionally, it elevated expression of Nrf2 and HO‐1 (Figure S1C,D). These observations suggest that hydrogen directly shields neuronal cells.

## 4 Discussion

SD negatively impacts cognitive and memory abilities [26], incurring substantial healthcare and societal expenses. Currently, no fully effective therapies exist. Evidence indicates that oxidative stress and inflammatory responses are key contributors to SD‐associated cognitive and memory decline [27]. SD initiates oxidative stress and inflammation [28], leading to insufficient free radical clearance, neuronal damage, and enhanced release of inflammatory cytokines—all independent risk factors for chronic diseases [29]. Consequently, interventions targeting oxidative stress and inflammation after SD are urgently required [30].

Previous work suggested that H_2_ inhalation improves sleep quality and cognition in patients with sleep disorders, supporting the concept that H_2_ confers these advantages through selective antioxidant action. Existing studies confirm that H_2_ can precisely stimulate antioxidant pathways while inhibiting pro‐oxidative stress pathways [31]. To explore this, an SD mouse model was established, and the consequences of H_2_ inhalation were assessed using the Morris water maze, NOR, and related tests. Hippocampal neuron morphology and brain tissue inflammatory cytokine levels were examined in parallel.

The Nrf2 signaling cascade is one of the most crucial cellular defense systems against oxidative stress [32]. Nrf2 chiefly exerts its antioxidant effects by activating downstream antioxidant enzymes [33]. The Nrf2/HO‐1 signaling axis fulfills a vital role in the oxidative stress response [32], contributing to anti‐inflammatory, antioxidant, and anti‐apoptotic processes, thereby representing a key therapeutic target for oxidative damage [30]. Notably, regulation of Nrf2 by sleep‐related genes implies that sleep may mediate antioxidative and anti‐inflammatory effects through modulation of Nrf2 signaling [32]. Therapeutic approaches focusing on this pathway have proven effective in reducing cognitive and memory deficits [34].

Molecular H_2_ exhibits significant biological activity [20], with distinctive features such as high tissue permeability and rapid diffusion, enabling it to cross the blood–brain barrier and directly modulate mitochondrial and cellular oxidative stress responses [35]. As a potent antioxidant, H_2_ activates the Nrf2/HO‐1 pathway, providing neuroprotection against diverse injuries [31]. Experimental evidence confirms the anti‐inflammatory, antioxidant, and neuroprotective properties of H_2_, including mitigation of radiation‐induced cognitive decline and hippocampal damage [36]. In rodent models of sepsis and SD, administration of H_2_‐rich water or H_2_ improves cognitive impairment linked to brain injury [37]. However, the precise neuroprotective mechanisms remain incompletely understood. Current literature proposes selective antioxidant activity as H_2_'s primary mode of action [38] though specific molecular pathways need further clarification. Growing evidence identifies the Nrf2 pathway as central to H_2_'s antioxidant effects [39]. with demonstrated critical roles in H_2_ therapy for sepsis [15]. Chen et al. [40] established that H_2_ modulates microglial polarization from M1 to M2 phenotype via Nrf2 activation, protecting neurons against lipopolysaccharide‐activated microglial damage through combined anti‐inflammatory and neuroprotective actions involving modulation of the TLR4/NF‐κB signaling pathway. Recent studies have shown that H_2_ suppresses the release of inflammatory cytokines such as IL‐1β, IL‐6, and TNF‐α, thereby alleviating tissue inflammation [21].

These outcomes demonstrate that inhaling H_2_ can relieve cognitive problems in sleep‐deprived mice by activating the Nrf2/HO‐1 pathway, thus reducing oxidative stress and inflammation. SD impairs free radical scavenging, inducing oxidative stress and inflammation that ultimately cause neuronal damage and cognitive dysfunction. Consistent with prior reports, the model group displayed significant cognitive impairment, pronounced neuronal injury, and abnormal levels of oxidative and inflammatory markers.

This study possesses certain limitations. First, reliance exclusively on mouse models underscores the need for clinical trials to verify H_2_'s efficacy in humans. Second, optimal treatment parameters (e.g., dose and timing) for H_2_ intervention require systematic investigation. Furthermore, while this research concentrated on clarifying the role of the Nrf2/HO‐1 signaling pathway, H_2_ may produce its antioxidative effects by regulating additional pathways. Future work should address these gaps to strengthen the theoretical foundation and clinical potential of H_2_ therapy for SD‐related cognitive impairment.

## 5 Conclusion

This investigation reveals that H_2_ inhalation effectively mitigates cognitive deficits in sleep‐deprived mice, likely by activating the Nrf2/HO‐1 signaling cascade to reduce oxidative stress and inflammation. These insights offer fresh perspectives for developing preventive and therapeutic strategies against SD‐induced cognitive dysfunction. Although H_2_ represents a promising therapeutic candidate, further research is required to validate and optimize its clinical use.

## Author Contributions

Conceptualization: QiFan Xiao; data curation: ShiRui Zhou and Bin Tang; formal analysis: QiFan Xiao and ShiRui Zhou; investigation: YuQing Zhu; methodology: Bin Tang and YuQing Zhu; writing – original draft preparation: QiFan Xiao; writing – review and editing: YuQing Zhu. All authors have read and agreed to the published version of the manuscript.

## Funding

This work was supported by National High Level Hospital Clinical Research Funding (2023‐NHLHCRF‐YYPPLC‐ZR‐23).

## Ethics Statement

All animal experiments were complied with the ARRIVE guidelines and performed in accordance with the National Institutes of Health Guide for the Care and Use of Laboratory Animals. The experiments were approved by the Institutional Animal Care and Use Committee of China‐Japan Friendship Hospital (ZRDWLL230137).

## Conflicts of Interest

The authors declare no conflicts of interest.

## Supporting information


**Figure S1:** Hydrogen ameliorates neuronal injury in vitro. (A) Cell viability assessed by CCK‐8 assay; (B) Apoptosis detected by flow cytometry; (C, D) Nrf2 and HO‐1 expression analyzed by RT‐qPCR and Western blot. Data are presented as mean ± SD. *p < 0.05, **p < 0.01, ***p < 0.001, ****p < 0.0001.

## Data Availability

Data are available from the corresponding author on request.
