# Supplementary material for: Hydrogen‐Mediated Activation of the Nrf2/HO‐1 Signaling Pathway Improves Cognitive Impairment in Sleep‐Deprived Mice
Source: CNS Neurosci Ther. 2026 Feb 1;32(2):e70770. doi: 10.1002/cns.70770 (PMC12862102; doi:10.1002/cns.70770)
Supplement: Supplementary file 1 — Figure S1: Hydrogen ameliorates neuronal injury in vitro. (A) Cell viability assessed by CCK‐8 assay; (B) Apoptosis detected by flow cytometry; (C, D) Nrf2 and HO‐1 expression analyzed by RT‐qPCR and Western blot. Data are presented as mean ± SD. *p < 0.05, **p < 0.01, ***p < 0.001, ****p < 0.0001. [file CNS-32-e70770-s001.zip › Supplementary file for review and publication.docx]

**
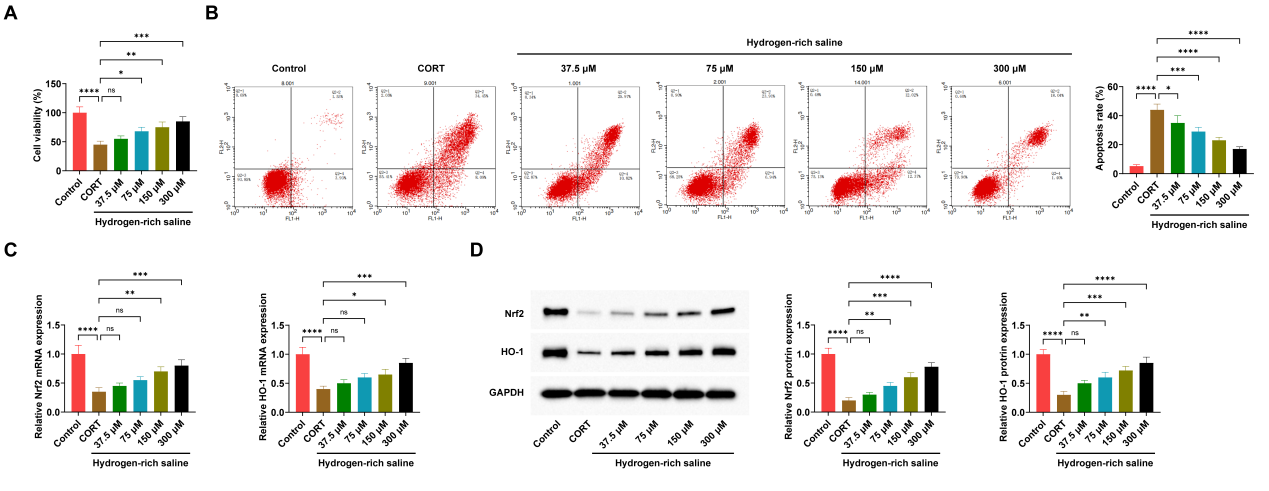
**

**Supplementary Figure 1. Hydrogen Ameliorates Neuronal Injury *In Vitro***

A: Cell viability assessed by CCK-8 assay; B: Apoptosis detected by flow cytometry; C, D: Nrf2 and HO-1 expression analyzed by RT‑qPCR and Western blot. Data are presented as mean ± SD. * *P* < 0.05, ** *P* < 0.01, *** *P* < 0.001, **** *P* < 0.0001.
